# PAFway: pairwise associations between functional annotations in biological networks and pathways

**DOI:** 10.1093/bioinformatics/btaa639

**Published:** 2020-07-17

**Authors:** Mahiar Mahjoub, Daphne Ezer

**Affiliations:** Department of Mathematics, University of Cambridge, Cambridge CB3 0WA, UK; The Alan Turing Institute, London NW1 2DB, UK; Royal Prince Alfred Hospital, Central Clinical School, University of Sydney, Sydney, NSW 2050, Australia; The Alan Turing Institute, London NW1 2DB, UK; Department of Statistics, University of Warwick, Coventry CV4 7AL, UK; Department of Biology, University of York, York, YO10 5NG, UK

## Abstract

**Motivation:**

Large gene networks can be dense and difficult to interpret in a biologically meaningful way.

**Results:**

Here, we introduce PAFway, which estimates pairwise associations between functional annotations in biological networks and pathways. It answers the biological question: do genes that have a specific function tend to regulate genes that have a different specific function? The results can be visualized as a heatmap or a network of biological functions. We apply this package to reveal associations between functional annotations in an *Arabidopsis thaliana* gene network.

**Availability and implementation:**

PAFway is submitted to CRAN. Currently available here: https://github.com/ezer/PAFway.

**Supplementary information:**

Supplementary data are available at *Bioinformatics* online.

## 1 Introduction

Biological networks can be very large, dense and difficult to visualize and interpret. PAFway is a tool to interpret large, dense biological networks in the context of functional annotations, such as gene ontology (GO). Some methods that analyse GO enrichment within networks, such as BiNGO (Maere *et al.*, 2005), operate by partitioning the network into clusters and then finding functional enrichment within each cluster.

Another family of methods, called topological network enrichment methods, utilize the structure of the network to find GO terms that are enriched in a network or sub-network (Mitrea *et al.*, 2013). The output of these algorithms is generally a ranked list of annotations, ordered by how much they are enriched in the network.

In contrast, PAFway finds *pairwise associations of functional annotations* in biological networks and pathways, which is calculated efficiently using the Fast Fourier Transform (FFT). The results can be illustrated either in the form of a heat map or as a network where the nodes in the graph are functional annotations. We apply this method to AraNet (Lee *et al.*, 2015), a gene network for *Arabidopsis thaliana*.

## 2 Materials and methods

The PAFway function takes as input a directed network, with or without edge weights, and a list all of the functional annotations associated with each node. We refer to each *edge type* as an ordered pair of functional annotations, representing the scenario where a gene with the first functional annotation regulates a gene with the second functional annotation. The output of PAFway is the probability of observing at least the observed number (or sum of edge weights) of each edge type, under a null model in which the functional annotations are randomly distributed in the network (after correcting for multiple hypothesis testing).

### 2.1 *P*-value of edge counts

Let us say that the relative frequency of the first functional annotation in the network is *p_a_* and the second is *p_b_*. The probability of observing an edge between annotations *a* and *b* is $p_{a,b}=p_{a}p_{b}$ if they are randomly distributed in the network. The probability of observing *n* edges between the first and second functional annotations in a network with *N* edges is determined by a binomial distribution:

(1) $$n∼B(N,p_{a,b})$$

This means that it is possible to determine the probability of observing at least *n* edges of a certain type by using the binomial test.

### 2.2 *P*-value of sum of edge weights

When a gene network contains edge weights, we calculate the sum of the edge weights of each edge type, to interrogate whether this value is higher than would be expected by chance. For two functional annotations *a* and *b*, let us define $z_{a,b}$ as the sum of the edge weights of edge type (*a*, *b*) in the network. Let us say that $c_{a,b}$ is the count of the number of edges of that type. $P(c_{a,b}=i)$ is the probability of observing exactly *i* edges of type (*a*, *b*) and $P(x\geq z_{a,b}|c_{a,b}=i)$ is the probability of observing a sum of edge weights greater than $z_{a.b}$ given that $c_{a,b}=i$. The probability of observing at least $z_{a,b}$ is:

(2) $$P(x\geq z_{a,b})=\sum_{i=1}^{N}P(c_{a,b}=i)P(x\geq z_{a,b}|c_{a,b}=i)$$

where *N* is the number of edges in the network. Note that, $P(x\geq z_{a,b})$ is the *P*-value. From the previous section, we see that $P(c_{a,b}=i)$ is the probability density function (pdf) of the binomial distribution $B(N,p_{a,b})$. $P(x\geq z_{a,b}|c_{a,b}=i)$ can be determined by a set of recursive functions described in Supplementary Section S1.1. These functions are convolutions and so can be expressed in terms of Fourier transforms and calculated efficiently using the FFT (see Supplementary Section S1.2).

## 3 Results

**Fig. 1.**
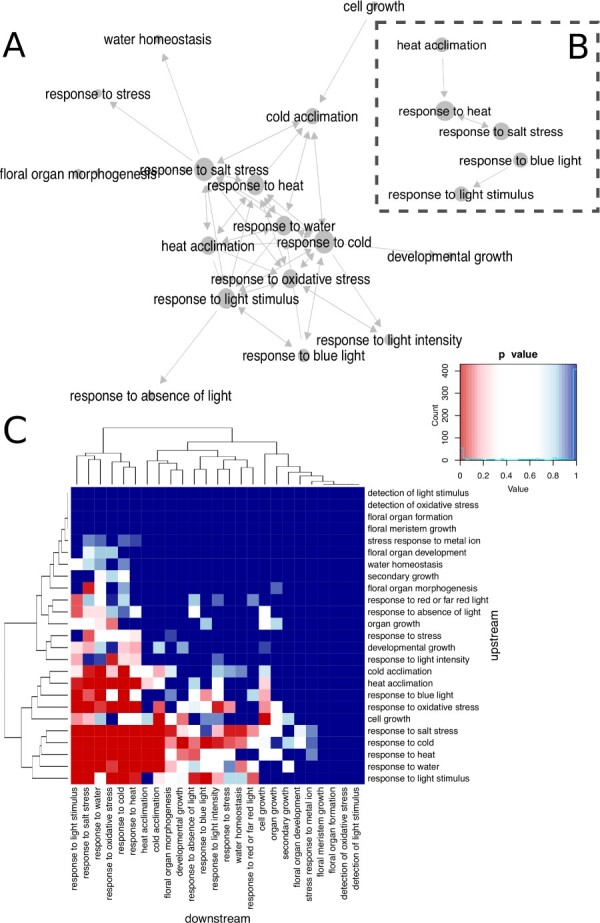
PAFway is applied to the AraNet gene network of *Arabidopsis thaliana*, either including (**A**) or ignoring (**B**) edge weights. Only edges with *P*-values < 0.05 are depicted. The network can also be represented as a heatmap (**C**), in this case depicting the same network as the one shown in (A)

PAFway produces a network of functional annotations, which can be depicted as a network (Fig. 1A and B) or a heatmap (Fig. 1C). This is shown for AraNet, a gene network for *Arabidopsis thaliana* (containing some co-expression-based edges) (Supplementary Section S2.1). We are not aware of any other tool for performing this precise task, but there are alternative packages that perform other kinds of complementary analyses of GO terms.

First, we compare the results of PAFway to a pairwise association score similar to the one proposed by Chitale *et al.* (2011) and Yerneni *et al.* (2018). Our method produces results that are consistent with this score, but with the added benefit of providing a *P*-value (Supplementary Section S2.2).

Next, we compare our results to those produced by NaviGO (Wei *et al.*, 2017), a tool that allows the user to calculate the similarity between pairs of GO terms, based on either semantic similarity (Lin, 1998; Resnik, 1999; Schlicker *et al.*, 2006) or how often they appear together in gene annotations (Chitale *et al.*, 2011), the scientific literature (Chitale *et al.*, 2011) and in physically interacting proteins (Yerneni *et al.*, 2018). We find that the strength of the correlation between our *P*-values and these metrics varies quite substantially based on whether edge weight information is incorporated in the model (Supplementary Section S2.3).

Finally, we cluster the AraNet network into communities, and visualize the GO terms within each community with both BiNGO (Maere *et al.*, 2005) and PAFway. We suggest that BiNGO can be used to help identify GO terms of interest whose relationships within the network could be further analysed with PAFway (Supplementary Section S2.4).

In conclusion, PAFway provides information that is complementary to these alternative methods, providing an innovative way to improve our understanding of large biological networks.

## Funding

Turing Research Fellowship under Engineering and Physical Sciences Research Council (EPSRC) grant [TU/A/000017]; EPSRC/Biotechnology and Biological Sciences Research Council (BBSRC) Innovation Fellowship [EP/S001360/1]; United Kingdom Research and Innovation (UKRI)/Turing Research Strategic Priority Fund [R-SPES-107].

*Conflict of Interest*: none declared.

## Supplementary Material

btaa639_supplementary_data
