# Supplementary material for: PAFway: pairwise associations between functional annotations in biological networks and pathways
Source: Bioinformatics. 2020 Jul 17;36(19):4963–4. doi: 10.1093/bioinformatics/btaa639 (PMC7750965; doi:10.1093/bioinformatics/btaa639)
Supplement: btaa639_supplementary_data [file btaa639_supplementary_data.pdf]

Supplementary Information for "PAFway: Pairwise Associations of Functional annotations in biological networks and pathways"

Mahiar Mahjoub[1,2,3] and Daphne Ezer[2,4,5][0000−0002−1685−6909]

[1] Department of Mathematics, University of Cambridge, Cambridge, UK
[2] The Alan Turing Institute, London, UK
[3] Royal Prince Alfred Hospital, Central Clinical School, University of Sydney, Sydney, Australia
[4] Department of Statistics, University of Warwick, Coventry, UK
[5] Department of Biology, University of York, York, UK
{daphne.ezer}@york.ac.uk

Table of Contents

Supplementary Information for "PAFway: Pairwise Associations of Functional annotations in biological networks and pathways" 1
Mahiar Mahjoub and Daphne Ezer
1 Supplementary Methods 2
1.1 P-value of sum of edge weights 2
1.2 Implementation with Fast Fourier Transform (FFT) 3
1.3 Notes about functional annotations that are associated with overlapping sets of genes 3
2 Supplementary Results 4
2.1 Biological evidence that the PAFway network in *Arabidopsis thaliana* is reasonable 4
2.2 Verification that method performs accurately 5
2.3 Comparison to NaviGO 7
2.4 Comparison to BiNGO 9
3 Discussion 10

1 Supplementary Methods

1.1 P-value of sum of edge weights

The recursursive functions refered to in the main text are:

$$P(x \geq z_{a,b}|c_{a,b} = i) = 1 - cdf_i(z_{a,b}|c_{a,b} = i) = 1 - \int_{-\infty}^{\infty} \text{pdf}_{i-1}(y)\text{cdf}_1(z_{a,b} - y)dy \quad (1)$$

and

$$\text{pdf}_k(x) = \int_{-\infty}^{\infty} \text{pdf}_{k-1}(y)\text{pdf}_1(x - y)dy \quad (2)$$

, where $\text{cdf}_1(x)$ and $\text{pdf}_1(x)$ are the cumulative density function and probability density function, respectively, for the distribution of edge weights, which we estimate with kernel density estimates (density function in R).

Equation (1) and (2) come from standard formulas for calculating pdfs and cdfs for sums of pairs of continuous random variables. In particular, for two probability distributions A and B:

$$\text{cdf}_{A+B}(x) = \int_{-\infty}^{\infty} \text{pdf}_A(y)\text{cdf}_B(x - y)dy \quad (3)$$

and

$$\text{pdf}_{A+B}(x) = \int_{-\infty}^{\infty} \text{pdf}_A(y)\text{pdf}_B(x - y)dy \quad (4)$$

In our case, we say that if $c_{a,b} = i$, then $z_{a,b}$ is constructed by sampling i values from the probability distribution of edge weights and summing these values together. This will give the same final outcome as would the following procedure:

1. Sample $i-1$ values from the distribution of edge weights and find the sum
2. Sample one more value from this distribution of edge weights
3. Find the sum of (1) and (2).

This means that when we calculate Equation 2, we can calculate $P(x \geq z_{a,b}|c_{a,b} = i)$ from $P(x \geq z_{a,b}|c_{a,b} = (i-1))$.

1.2 Implementation with Fast Fourier Transform (FFT)

Note that both the functions for $\text{pdf}_k(x)$ and $\text{cdf}_i(x)$ are convolutions of two functions; recall the definition of convolution:

$$\text{convolution}(f,g)(y) = \int_{-\infty}^{\infty} f(x)g(y-x)dx \tag{5}$$

This can be expressed in terms of Fourier transforms:

$$\int_{-\infty}^{\infty} f(x)g(y-x)dx = \text{Fourier}^{-1}(\text{Fourier}(f(x))\text{Fourier}(g(x))) \tag{6}$$

We estimate this with a discrete Fourier transform via the Fast Fourier Transform (FFT). This means that instead of calculating an integral, we sample $f(x)$ and $g(x)$ at m discrete points, forming the vectors $f[x]$ and $g[x]$ of length m. The discrete form of the convolution can be calculated by the following summation: $h[k] = \sum f[x]g[k-m-x]$ for all valid x, leading to a vector of $h[x]$ of length $2m$. In order to prevent the vector from expanding in size too much as we estimate $\text{pdf}_k(x)$, we drop the elements in the beginning and end of the vector that have values that are very close to zero (by default $f[x] < e - 05$).

1.3 Notes about functional annotations that are associated with overlapping sets of genes

Genes may have multiple functional annotations associated with them. For instance, it is often the case that *all* genes that have one GO term will also have a different specific GO term, because GO terms have a parent-child arrangements (e.g. all genes involved in 'blue light sensing' are also 'light sensing'). In addition, sometimes two related GO terms may be associated with largely overlapping sets of genes (like 'salt stress' and 'drought stress').

This does not directly impact the calculation of p-values in PAFway, but it would potentially impact how we interpret the results from a biological perspective and could be used to filter out edges by biological importance . The aim of PAFway is to evaluate whether an edge between two functional annotations

occurs more frequently than would be expected by a null model in which edges are randomly distributed in the network. In this scenario, it does not matter how much overlap there is between the functional annotations. Intuitively this makes sense: Let's say you randomly select two words from a book and you want to calculate the probability that the first word you select contains the letter 'a' and the second word you select contains the letter 'b'. The expected probability of this happening is $p_a * p_b$, and it does not depend on whether the letters 'a' and 'b' tend to appear together in the same word. This means that the calculation in PAFway is correct, as is.

However, this also means that the frequencies of pairs of edges are correlated with one another. For instance, if GO term 'a' is enriched upstream of 'b', and many genes that have GO term 'a' are also labelled with GO term 'c', then there may also be an enrichment for edges between genes containing the GO terms 'c' and 'b'. If there is an overlap between GO terms 'b' and 'd', then there may also be an enrichment for edges between 'c' and 'd'. Biologists may be less interested in an enrichment between GO terms 'c' and 'd' if this enrichment is completely explained by the enrichment between GO terms 'a' and 'b'.

For this reason, we may want to know whether we observe more edges between genes containing the GO terms 'c' and 'd', given (i) the co-occurrence of functional annotations 'a' and 'c' (ii) the co-occurrence of functional annotations 'b' and 'd' (iii) the frequency of edges containing 'a' and 'b'. In particular, let $f_{i,j}$ be the number of times we observe an edge between genes with functional annotations i and j. Let $p(j|i)$ be the probability of a gene having the functional annotation j given that it has the functional annotation i. We use the notation $!i$ to signify *not* i. We can calculate the expected value of $f_{c,d}$ using the following formula:

$$\begin{aligned}\mathbb{E}(f_{c,d}) = & f_{a,b}p(c|a)p(d|b)+ \\ & f_{!a,b}p(c|!a)p(d|b)+ \\ & f_{a,!b}p(c|a)p(d|!b)+ \\ & f_{!a,!b}p(c|!a)p(d|!b)\end{aligned}$$

Then, we can compare the observed count with the expected count: $f_{c,d}/\mathbb{E}(f_{c,d})$. We have added the function to the PAFway package to be able to calculate this value for every possible pair of functional annotations.

2 Supplementary Results

2.1 Biological evidence that the PAFway network in *Arabidopsis thaliana* is reasonable

The main output of PAFway is a network of functional annotations, which is shown in Fig. 1 in the main text for an *Arabidopsis thaliana* network. These results are consistent with what we would expect: for instance, cold acclimation

is known to be related to response to salt stress [5], response to salt stress is known to be related to water homeostasis [1], and response to cold is related to developmental growth [7]. Also, by definition, heat acclimation will impact the plant's response to heat. Some edges are more surprising and warrant further investigation, such as whether cell growth impacts cold acclimation.

2.2 Verification that method performs accurately

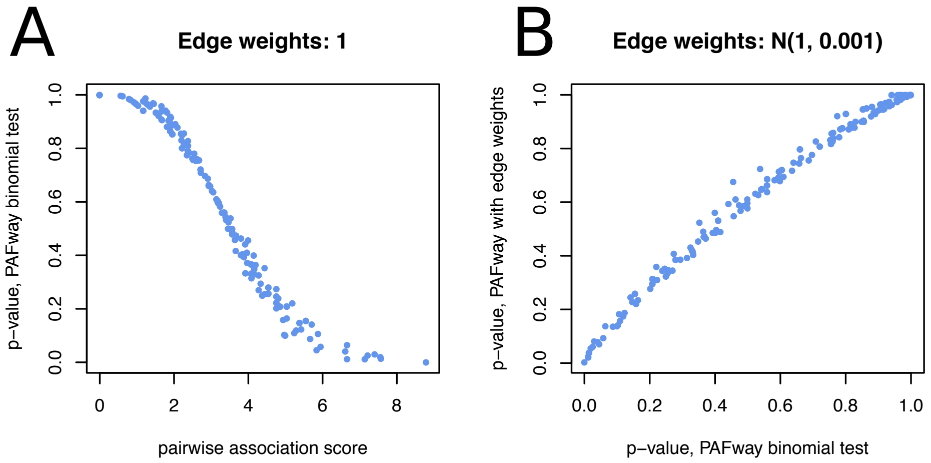

Fig. 1. Verification that PAFway produces reasonable outputs. (A) PAFway produces results that are consistent with a pairwise association score. (Note that a higher pairwise association suggests a lower p-value) (B) The FFT-based approach for estimating p-values with edge weights produces results that are consistent with a binomial test, when the edge weights do not vary substantially from 1.

First, we compare the results of PAFway to a pairwise association score that is similar to the one proposed by [3, 14], where $S(a, b)$ is the association score between GO term a and b, $p_{a,b}$ is the proportion of edges in the network that go from GO term a and b, and p_a and p_b are the proportion of genes in the network that have GO terms a and b, respectively.

$$S(a, b) = \frac{p_{a,b}}{p_a p_b}$$

Values greater than 1 indicate that the edge type is enriched in the network, while values less than 1 indicate that the edge type is under-represented in the network. However, this metric does not indicate whether the over- or under-representation in the network is significant. Additionally, this method does not enable us to incorporate information about edge weights.

We find that the results are highly correlated to one another when the edge weights are one (Fig 1(A)) or close to one (Fig 1(B), edge weights sampled from

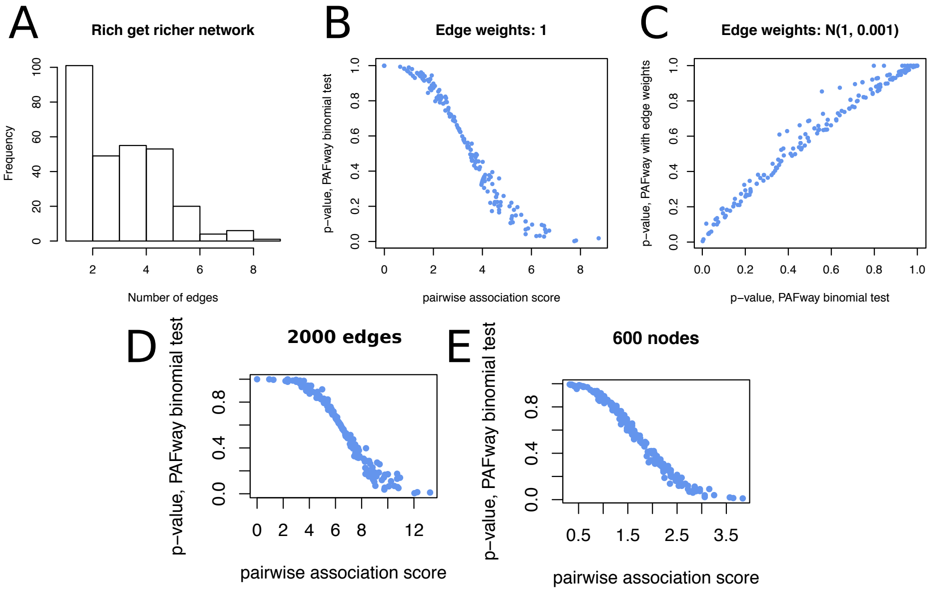

Fig. 2. Verification that PAFway produces reasonable outputs for a Rich-Get-Richer (RGR) network. (A) A random RGR network was made, by making the probability of generating a new edge associated with a node be proportional to the current number of edges associated with that node. This is the distribution of edge weights that were produced. (B) This is the equivalent figure to Fig. 1(A) for this RGR network. (C) This is the equivalent figure to Fig. 1(B) for this RGR network. (D) A simulated network was constructed as in Fig. 1(B), but the number of edges was increased from 1000 edges to 2000 edges. The pairwise association score that corresponds to a p-value of 0.05 increases in this circumstance. (E) The number of nodes (i.e. genes) in the simulated network in Fig. 1(B) was increased from 300 nodes to 600 nodes. In this circumstance, the pairwise association score that corresponds to a p-value of 0.05 decreases. These last two scenarios illustrate that the p-value associated with a particular pairwise association score varies, depending on the network.

a Gaussian with a mean of 1 and standard deviation of 0.001), in a random network with 300 genes, 1000 edges and 14 functional annotations and scale-free (Fig 2) networks.

We also varied the size and density of the simulated network, in order to evaluate how it would impact the relationship between pairwise association scores and p-values. We find that doubling the number of edges (Fig 2C) and/or doubling the number of genes (Fig 2D) impact which values of the pairwise association scores map to p-values of 0.05. This suggests that there does not appear to be a simple 'rule-based' approach for assigning p-values directly based on the pairwise-association scores.

2.3 Comparison to NaviGO

NaviGO [13] is a tool that allows the user to calculate the similarity between pairs of GO terms, based on either semantic similarity [11, 12, 6] or how often they appear together in gene annotations [3], the scientific literature [3], and in physically interacting proteins [14].

The GO consortium have organised all GO terms into three non-overlapping directed acyclic graphs (DAG) that describe their relationships [4]; for instance, there are many different GO terms describing specific responses to many kinds of stress, but these all fall under the GO term 'response to stress' and so are connected to this GO term in the DAG. Semantic similarity scores reference the structural relationships between GO terms within this DAG. For instance, the Resnik score is calculated by first finding the lowest common ancestor of the two GO terms and taking $-log$ of the proportion of genes that have that annotation [11]. The Lin score [6] is the information content of the lowest common ancestor and the relevance semantic similarity (RSS) score [12] weighs the information content by the probability of observing a common ancestor by chance. On the other hand, the Co-occurrence Association Score (CAS), Pubmed Association Score (PAS), and Interaction Association Score (IAS) measure the relative enrichment of two terms co-occurring in gene annotations (CAS), the literature (PAS), and in protein interaction networks (IAS) [3, 14]. These three metrics use a similar ratio as the pairwise association score $S(a, b)$ described in the previous section.

To evaluate how our results using PAFway are correlated to these other metrics, we began by selecting a number of GO terms that are relevant to the main research interests of our lab group. These included GO terms associated with sensing and acclimating to light, temperature and water, stress response, growth, and flower development. Then, we calculated the following values for the AraNet gene network: (i) various pairwise GO comparison metrics using NaviGO (Fig 3) and (ii) the PAFway p-values, with and without edge weights.

For all GO term pairs where there was at least one connection in AraNet, we compared their PAFway associated p-values with the scores produced by the three semantic similarity metrics (Resnik, Lin, and RSS) and the three pairwise association scores (CAS, PAS, and IAS). Interestingly, there was more of a correlation between semantic similarity measures and the results from PAFway when

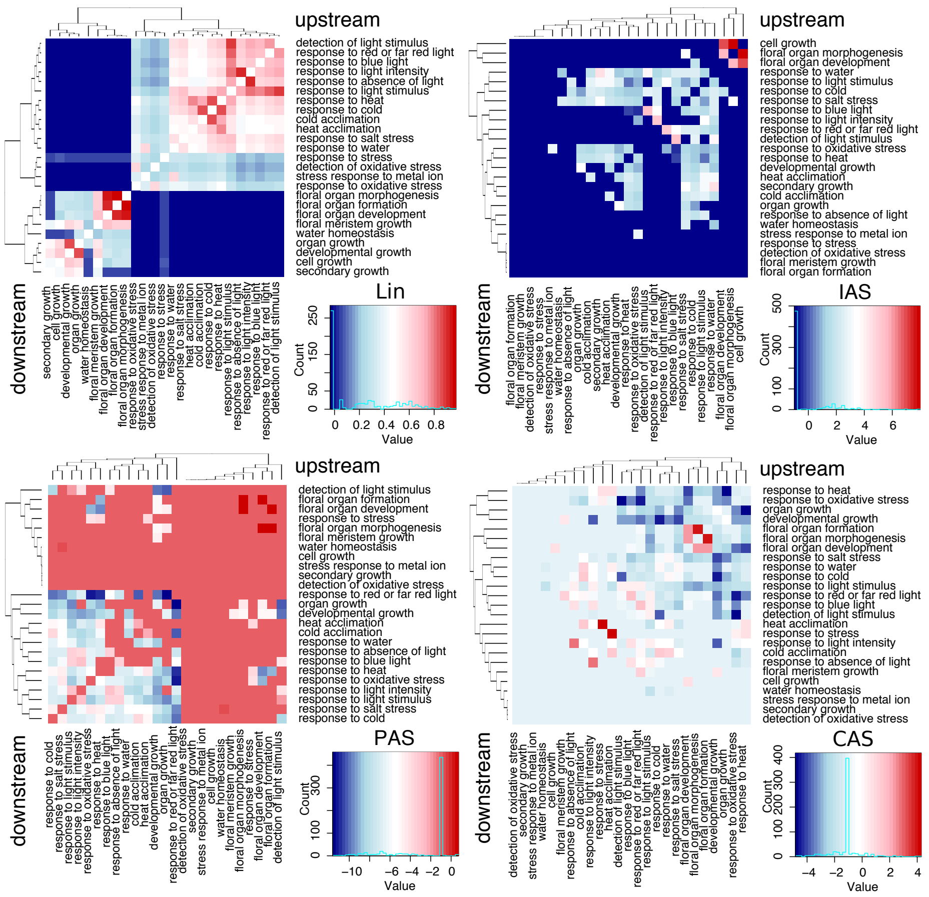

Fig. 3. Comparison of selected GO terms under semantic similarity (Lin) and other pairwise-association scores (PAS, CAS, IAS). Resnik and RSS are not shown because they produce results that are extremely similar to Lin.

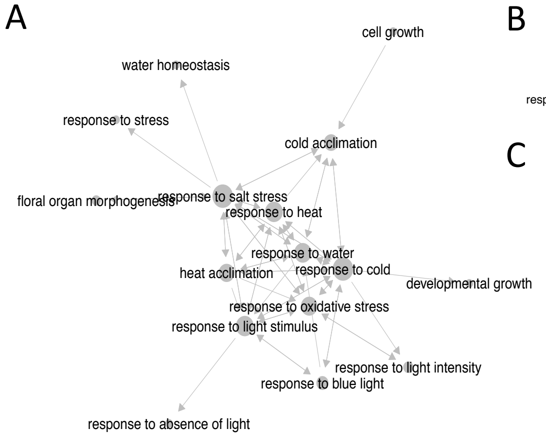

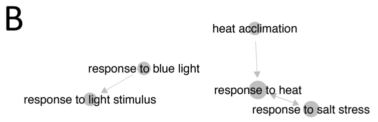

C

P-values of correlation after Bonferroni correction	PAFway, no edge weights	PAFway, with edge weights
Resnik	2.25e-05	0.048
Lin	1.31e-07	0.077
RSS	1.13e-07	0.058
ln(CAS)	0.138	0.004
ln(PAS)	0.025	0.004
ln(IAS)	1	0.360

Fig. 4. Comparison of PAFway to other metrics that compare pairs of GO terms. (A) PAFway summary network of AraNet, when edge counts are considered. (B) PAFway summary network of AraNet, when edge weights are considered. (C) Comparison between PAFway p-values and other metrics in NaviGO.

edge counts (not edge weights) were considered. On the other hand, when edge weights were considered, the results were more significantly associated with the CAS and PAS scores, which measure co-occurrence of GO terms in gene annotations and in the literature. This suggests that while PAFway provides results that are somewhat consistent with other metrics of pairwise GO term similarities, it also provides new insights that cannot be directly gleaned by these metrics.

2.4 Comparison to BiNGO

BiNGO [8] is a popular cytoscape plugin that is similar to PAFway, in that it depicts the GO terms as a network. More specifically, it identifies GO terms that are enriched in clusters (or communities) within a network, and then depicts these enriched GO terms as a network, where edges represent the underlying network structure of the GO DAG. For instance, *phosphate metabolic process* points to *dephosphorylation* because dephospholylation is a type of phosphate metabolic process. Non-enriched GO terms are also included in the BiNGO network if they are required to make it fully-connected. The BiNGO GO term network differs from the PAFway network, because PAFway edges relate to GO term associations within the original biological network, rather simply reflecting the GO term definitions. In this section, we demonstrate the differences between these networks and illustrate how they can work together to improve our ability to interpret large biological network.

First, we find communities within the AraNet network, using spinglass community detection[10] (5 runs), followed by consensus clustering with K-means. We found 131 unique communities, but 20 of these communities were substantially larger than the others, so we focus on these. Fig 5(A) shows some of the key words found among the GO terms in a random selection of the 20 largest communities.

Fig 5(A) shows the BiNGO network that results from the analysis of the first of these communities (the word cloud in the top left). The full networks output of BiNGO is shown in the upper right panel of Fig. 5(B), but this is too large to be easily visualised, so we include a zoomed-in version of an interesting sub-network related to amino acid biosynthesis.

For our PAFway analysis, we decided to focus on GO terms that were highly enriched in each community (p-value$< 1e-10$) and that were not commonly found to be enriched among the top 20 largest communities (were found to be enriched in less than four of the 20 other large communities). The results of PAFway (with no edge weights) are shown in Fig. 5(C-D) as for the BiNGO network in Fig. 5(B) and for a sample of other sub-networks in Fig. 6. These can be expressed as either a network (Fig. 5(C)) or a heatmap (Fig. 5(D)). From this, we learn additional information that we cannot see from the BiNGO network. For instance, the close relationships between the biosynthesis pathways of the hydrophobic amino acids leucine, isoleucine and valine is much clearer from the PAFway output.

However, since BiNGO and PAFway provide different information, they can be used hand-in-hand to analyse a network. In particular, BiNGO can be used as an initial tool to identify GO terms of interest, which can be investigated in the context of the network structure using PAFway.

3 Discussion

This manuscript introduces a method for condensing a large hairball network into a network that links functional annotations or GO terms. We show that it provides complementary biological insights to other methods for comparing pairs of GO terms [13, 3, 14, 11, 12, 6]. It also provides complementary information to BiNGO, a popular way of visualising GO terms in a network format [8].

PAFway produces very different outputs depending on whether edge weights are considered. This is because in the AraNet network, the distribution of edge weights has a long-tail, so a small number of edges that have a very high weight play a more important role in the resulting network. On one hand, this is a good thing because these edges are much more likely to be biologically meaningful, so they should be weighed more heavily. On the other hand, the results may be less robust, because they depend on the values of a few edges. We recommend using both techniques and comparing the results.

Another consideration is whether to display the results of PAFway as a network or heatmap. The heatmap allows us to visualise the distribution of p-values that do not make the cut-off, so it can provide complementary information. For

instance, it is often easier to see 'cluster'-like behaviours from the heatmap visualisation. However, it may be tempting to add *meaning* to spurious relationships between GO terms that are not significantly linked when observing the heatmap visualisation, so in many ways the network representation is preferable.

Another issue is that most edges in these large hairball networks are likely to be false positives. Indeed, many validation studies find that at most half of the edges are direct targets [2, 9]. While having many false positives will decrease the sensitivity of our method to detect relationships between functional annotations, this should not effect the specificity of the method if the false positive edges have randomly distributed gene function annotations.

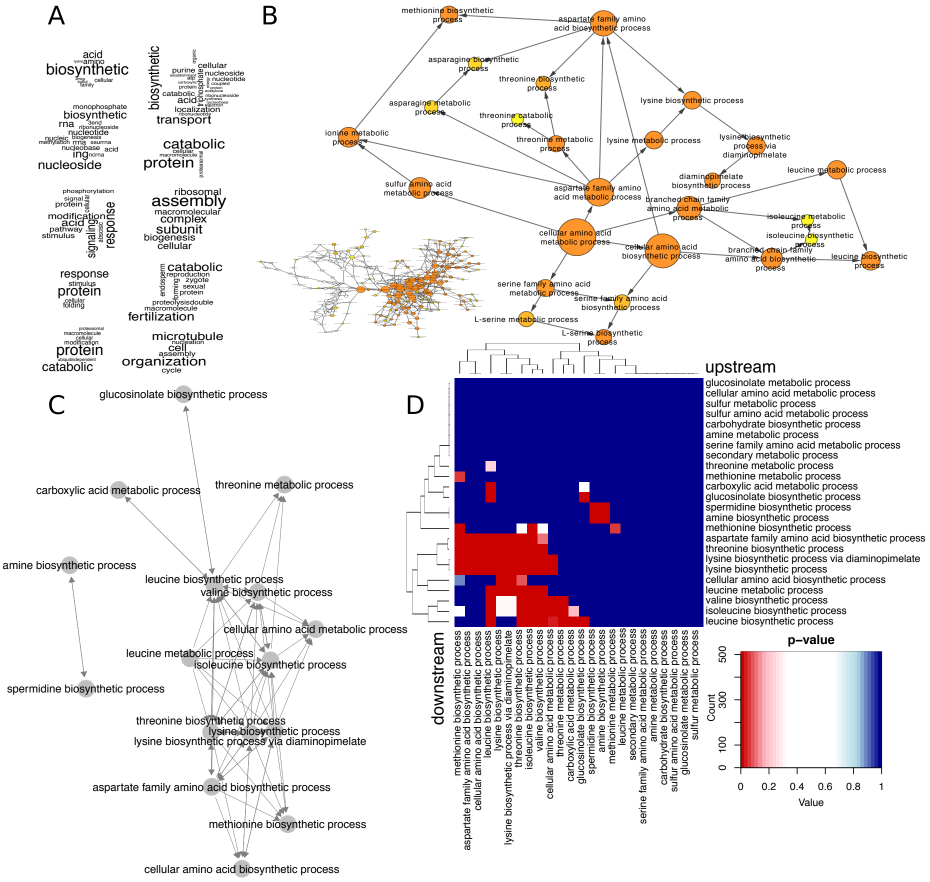

Fig. 5. Comparison of BiNGO and PAFway outputs. (A) Word clouds of key words among GO terms that are significantly enriched in a random selection of 10 of the 20 largest communities in AraNet. (B) BiNGO network of one community (the one represented by the top-left word cloud in (A). The full network is shown in the bottom left portion of this panel, but a zoomed-in sub-network that is related to amino acid biosynthesis and metabolism is also shown. (C) This is the network output of PAFway for the community shown in (B). A heatmap representation of this PAFway network is shown in (D).

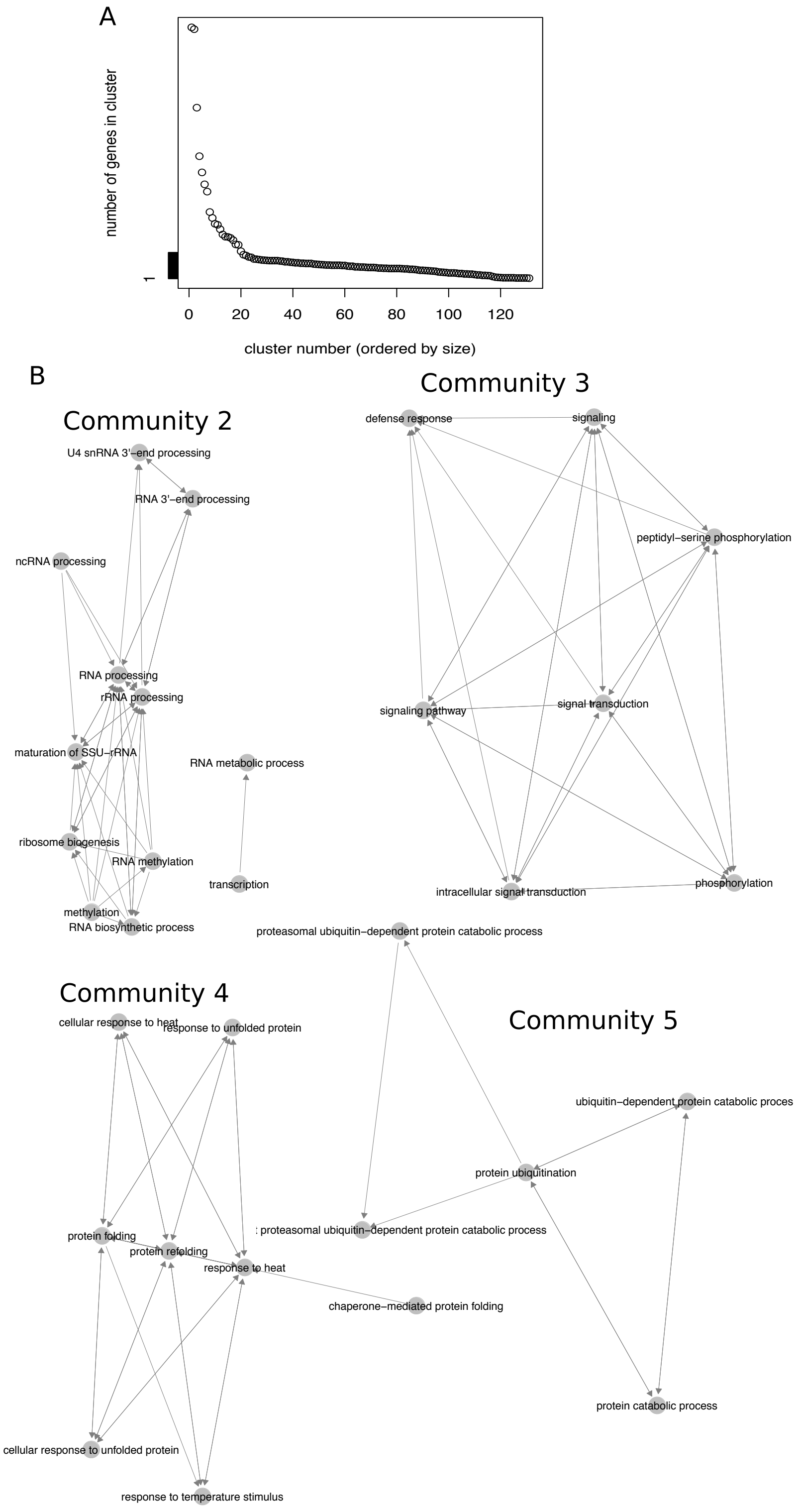

Fig. 6. Data from more communities (A) The size distribution of the communitities (B) The PAFway network for a few additional communities, ignoring edge weights, and correcting for multiple hypothesis testing with Bonferonni. The other 15 largest networks are available on the Github.

References

1. Boursiac, Y., Chen, S., Luu, D.T., Sorieul, M., Van Den Dries, N., Maurel, C.: Early effects of salinity on water transport in Arabidopsis roots. Molecular and cellular features of aquaporin expression. Plant Physiology (2005). https://doi.org/10.1104/pp.105.065029
2. Brooks, M.D., Cirrone, J., Pasquino, A.V., Alvarez, J.M., Swift, J., Mittal, S., Juang, C.L., Varala, K., Gutiérrez, R.A., Krouk, G., Shasha, D., Coruzzi, G.M.: Network Walking charts transcriptional dynamics of nitrogen signaling by integrating validated and predicted genome-wide interactions. Nature Communications (2019). https://doi.org/10.1038/s41467-019-09522-1
3. Chitale, M., Palakodety, S., Kihara, D.: Quantification of protein group coherence and pathway assignment using functional association. BMC Bioinformatics (2011). https://doi.org/10.1186/1471-2105-12-373
4. Gene Ontology Consortium: The Gene Ontology (GO) database and informatics resource. Nucleic Acids Research (2004). https://doi.org/10.1093/nar/gkh036
5. Kim, M.H., Sato, S., Sasaki, K., Saburi, W., Matsui, H., Imai, R.: COLD SHOCK DOMAIN PROTEIN 3 is involved in salt and drought stress tolerance in Arabidopsis. FEBS Open Bio (2013). https://doi.org/10.1016/j.fob.2013.10.003
6. Lin, D.: An Information-Theoretic Definition of Similarity. In: ICML (1998)
7. Liu, Y., Tabata, D., Imai, R.: A Cold-Inducible DEAD-box RNA helicase from arabidopsis thaliana regulates plant growth and development under low temperature. PLoS ONE (2016). https://doi.org/10.1371/journal.pone.0154040
8. Maere, S., Heymans, K., Kuiper, M.: BiNGO: A Cytoscape plugin to assess over-representation of Gene Ontology categories in Biological Networks. Bioinformatics (2005). https://doi.org/10.1093/bioinformatics/bti551
9. Marbach, D., Costello, J.C., Küffner, R., Vega, N.M., Prill, R.J., Camacho, D.M., Allison, K.R., Consortium, D., Kellis, M., Collins, J.J., Stolovitzky, G.: Wisdom of crowds for robust gene network inference. Nature methods **9**(8), 796–804 (jul 2012). https://doi.org/10.1038/nmeth.2016
10. Reichardt, J., Bornholdt, S.: Statistical mechanics of community detection. Phys. Rev. E **74**, 016110 (Jul 2006). https://doi.org/10.1103/PhysRevE.74.016110, https://link.aps.org/doi/10.1103/PhysRevE.74.016110
11. Resnik, P.: Semantic Similarity in a Taxonomy: An Information-Based Measure and its Application to Problems of Ambiguity in Natural Language. Journal of Artificial Intelligence Research (1999). https://doi.org/10.1613/jair.514
12. Schlicker, A., Domingues, F.S., Rahnenführer, J., Lengauer, T.: A new measure for functional similarity of gene products based on gene ontology. BMC Bioinformatics (2006). https://doi.org/10.1186/1471-2105-7-302
13. Wei, Q., Khan, I.K., Ding, Z., Yerneni, S., Kihara, D.: NaviGO: Interactive tool for visualization and functional similarity and coherence analysis with gene ontology. BMC Bioinformatics (2017). https://doi.org/10.1186/s12859-017-1600-5
14. Yerneni, S., Khan, I.K., Wei, Q., Kihara, D.: IAS: Interaction Specific GO Term Associations for Predicting Protein-Protein Interaction Networks. IEEE/ACM transactions on computational biology and bioinformatics (2018). https://doi.org/10.1109/TCBB.2015.2476809
